# Gut frailty in chronic heart failure: clinical determinants and distinct microbial signatures

**DOI:** 10.3389/fcvm.2026.1779873

**Published:** 2026-06-19

**Authors:** Mengdie Hu, Jiahui Xiang, Xinyi Hou, Yuxuan Shao, Qin Peng, Chaofeng Li

**Affiliations:** 1 Nanjing University of Chinese Medicine, Nanjing, China; 2 Eight-year Medical Doctor Program, Chinese Academy of Medical Sciences and Peking Union Medical College, Beijing, China; 3 The Second Hospital of Nanjing, Affiliated to Nanjing University of Chinese Medicine, Nanjing, China

**Keywords:** chronic heart failure, dietary pattern, dysbiosis, gut frailty, gut microbiota

## Abstract

**Background:**

Gut frailty is a critical yet under-recognized sub-phenotype in chronic heart failure (CHF) that may exacerbate systemic inflammation. This study aimed to identify risk factors for gut frailty in CHF and characterize associated gut microbiome alterations.

**Methods:**

In this cross-sectional study of 270 CHF patients, gut frailty was defined as a Gastrointestinal Symptom Rating Scale score of ≥3. Clinical determinants were identified via multivariable logistic regression. 16S rRNA sequencing was performed on a balanced sub-cohort (*n* = 60) to analyze microbial diversity, taxonomy, and predicted metabolic functions.

**Results:**

A meat-rich diet independently increased gut frailty risk (OR = 3.995; 95% CI: 1.107–16.110; *P* = 0.041), while vegetarian adherence was protective (OR = 0.148; 95% CI: 0.024–0.883; *P* = 0.035). Alpha-diversity was significantly reduced across all indices (Observed ASVs, Shannon, Chao1, ACE; all *P* < 0.0001), with distinct beta-diversity clustering (ANOSIM R = 0.2766, *P* = 0.001). The frail phenotype harbored fewer unique ASVs (76 vs. 142) and exhibited depletion of short-chain fatty acid producers (*Faecalibacterium*, *Coprococcus*) alongside enrichment of pathobionts and trimethylamine-producers (*Lachnoclostridium*, *Enterobacteriaceae*). Functionally, the microbiome shifted toward stress-responsive xenobiotic biodegradation, notably cytochrome P450 (log_2_ fold change = 2.82).

**Conclusion:**

Gut frailty in CHF constitutes a distinct syndrome modulated by diet and characterized by profound dysbiosis and metabolic reprogramming. Targeting the gut-heart axis through nutritional or microbiome-directed interventions may offer a novel strategy to mitigate frailty in heart failure.

## Introduction

Chronic heart failure (CHF) represents the terminal pathway of various cardiovascular pathologies, characterized by the heart's inability to meet systemic metabolic demands (1). Beyond traditional hemodynamic alterations, the “gut hypothesis” has emerged as a critical pathophysiological driver of CHF progression. This paradigm posits that splanchnic hypoperfusion and mucosal congestion compromise the intestinal barrier, facilitating bacterial translocation and systemic inflammation (2–4). Specifically, the leakage of luminal lipopolysaccharides (LPS) into the systemic circulation, termed metabolic endotoxemia, activates Toll-like receptor 4 (TLR4) signaling on immune cells and cardiomyocytes. This cascade triggers the release of pro-inflammatory cytokines, such as TNF and IL-6, which directly suppress myocardial contractility and promote cardiac fibrosis (2). Consequently, gastrointestinal (GI) symptoms, ranging from early satiety to abdominal distension, are highly prevalent yet frequently underappreciated clinical manifestations in patients with advanced CHF.

As the CHF population ages, systemic frailty frequently supervenes, creating a vicious cycle of multisystem decline and worsened prognosis (5). The digestive system is acutely susceptible to this age-related degeneration, manifesting as reduced motility, diminished enzymatic secretion, and impaired nutrient absorption (6). In patients with CHF, these physiological changes are severely exacerbated by disease-specific factors, including splanchnic congestion, neurohormonal activation, and polypharmacy, which collectively accelerate intestinal barrier dysfunction and disrupt gut ecology (2, 4). These alterations not only produce symptomatic distress, such as functional dyspepsia and constipation, but also predispose patients to malnutrition and sarcopenia. While digestive aging is increasingly recognized as a catalyst for systemic frailty, its specific intersection with CHF pathophysiology remains poorly defined (7).

To encapsulate this distinct gastrointestinal vulnerability, the concept of “gut frailty” has recently been introduced. Initially characterized in geriatric medicine, gut frailty denotes a pathological, age-related decline in GI function—encompassing diminished mucosal integrity, impaired motility, and dysbiosis—that precipitates clinical symptoms and drives systemic frailty (8). Operationally, gut frailty can be assessed using the Gastrointestinal Symptom Rating Scale (GSRS), a validated 15-item instrument evaluating five symptom domains (reflux, abdominal pain, dyspepsia, diarrhea, and constipation). In clinical research, a GSRS total score of ≥3 indicating at least “mild discomfort” serves as a practical screening threshold that consistently associated with clinically meaningful GI dysfunction across metabolic, nephrological, and post-surgical populations (9–11).

Although the mechanistic framework of the gut-heart axis is expanding, the prevalence, clinical determinants, and microbial underpinnings of gut frailty within the specific context of CHF remain uncharacterized (12). Bridging this knowledge gap is essential for developing targeted, non-hemodynamic interventions to interrupt the gut-heart-frailty cycle. Therefore, this study aimed to investigate the prevalence and clinical risk factors of gut frailty (defined as GSRS ≥ 3) in a CHF cohort. Furthermore, utilizing 16S rRNA gene sequencing, we sought to characterize the specific gut microbiota signatures associated with this phenotype, with the goal of identifying microbial biomarkers and novel therapeutic targets for mitigating gut frailty in heart failure.

## Materials and methods

### Participants and procedures

A total of 270 patients diagnosed with CHF were sequentially recruited from the Departments of Cardiology and Geriatrics at a tertiary hospital in China between September 2024 and May 2025. To minimize potential confounding effects arising from critical illness and associated medical interventions, strict inclusion and exclusion criteria were applied during patient enrollment, the details of which are fully elaborated in the Supplementary Method. A pilot study (*n* = 35) was conducted to calculate the required sample size of enrolled participants, as the details also listed in Supplementary Method. Every enrolled participant completed a comprehensive, enhanced paper-based questionnaire utilized to systematically investigate several lifestyle and health parameters, specifically dietary patterns, sleep duration, and exercise habits.

To achieve a robust, multidimensional evaluation of the participants' overall health status and frailty, five internationally recognized assessment tools were additionally employed. The Fried Frailty Phenotype (FP) was used to define frailty as a distinct biological syndrome characterized by decreased physiological reserve and resistance to stressors. The GSRS served as a widely utilized patient-reported outcome measure to quantitatively assess the severity and frequency of common gastrointestinal symptoms. Furthermore, the Oral Frailty Index-8 (OFI-8) functioned as a simplified screening tool to evaluate oral frailty, a cumulative decline in oral functions that precedes and predicts adverse systemic health outcomes. The Mini Nutritional Assessment-Heart Failure (MNA-HF) screening tool was employed to accurately assess nutritional status and the specific risk of malnutrition in heart failure patients. Finally, the Patient Health Questionnaire-9 (PHQ-9), a concise, self-administered diagnostic and severity measure for major depressive disorder, was also utilized. The detailed investigative content and scoring methodologies for all five assessments are fully provided in the Supplementary Method.

Due to resource constraints, a focused sub-cohort of 60 patients was randomly selected to undergo 16S rRNA microbiota sequencing between September 2024 and December 2024. This specialized cohort was intentionally balanced, consisting of 30 patients diagnosed with gut frailty and 30 without frailty. All research procedures received ethical approval from the Medical Ethics Committee of Nanjing Second Hospital (Approval No. 2025-LS-ky-013). The study was rigorously conducted in strict adherence to the principles of the Declaration of Helsinki and the International Council for Harmonisation Good Clinical Practice (ICH-GCP) guidelines. Finally, written informed consent was obtained from all participants prior to their enrollment.

### Measurement of gut frailty

To assess the presence of gut frailty, the GSRS, a scale capable of comprehensively evaluating both upper and lower gastrointestinal tract symptoms, was utilized (13). The GSRS is a patient-reported outcome measure comprising 15 items across five distinct symptom clusters: reflux, abdominal pain, dyspepsia (indigestion), diarrhea, and constipation. Each item is rated on a 7-point Likert scale (ranging from 1, no discomfort, to 7, very severe discomfort) or, alternatively, scored from 0 (mildest) to 4 (most severe), yielding a total score where higher values reflect more severe symptoms. We defined impaired intestinal function (gut frailty) as a GSRS total score of ≥3. This threshold was selected based on its established clinical significance and validation in previous studies, which have linked scores of ≥3 to functional impairments (9–11). The scale has consistently demonstrated good internal consistency and reproducibility, particularly in Asian populations, contributing to the clinical elucidation of gut frailty.

### Identification of risk factors

To identify the demographic, clinical, and behavioral determinants of gut frailty in the CHF cohort, a two-step logistic regression analysis was performed. Initially, the potential risk factors, including continuous variables such as Age, Body Mass Index (BMI), Systolic Blood Pressure, Diastolic Blood Pressure, laboratory markers (C-Reactive Protein [CRP], White Blood Count [WBC], Hemoglobin, Total protein, Albumin, Triglycerides, Total Cholesterol (TC), High-Density Lipoprotein Cholesterol [HDL-C], Low-Density Lipoprotein Cholesterol [LDL-C]), and frailty scores (FP, OFI-8, MNA-HF, PHQ-9), were subjected to univariate logistic regression. Categorical variables, including gender, left ventricular ejection fraction (LVEF) status, New York Heart Association (NYHA) stage, comorbidity status (Diabetes mellitus, CHF duration), and various lifestyle factors (Alcohol consumption, Smoking, Residence, Education level, Sleep duration, Regular exercise, Sedentary time, Dietary preferences, Dietary taste, and Number of medications), were also included in this initial screening. To assess potential multicollinearity, the generalized variance inflation factor (GVIF) was calculated. The adjusted GVIF value was calculated as $GVIF^{1/(2\times Df)}$, where Df represents the degrees of freedom. Subsequently, variables demonstrating statistical significance (*P* < 0.05) in the univariate analysis were included in a multivariable logistic regression model. This final model aimed to identify the independent predictors of gut frailty.

### Stool sample collection and 16S rDNA sequencing

This retrospective study utilized archived stool samples sourced from the standardized biobank maintained by Nanjing Hospital of Chinese Medicine Affiliated to NJUCM. All specimens were originally collected prospectively following a unified clinical protocol to ensure high data quality. For collection, patients first voided urine to minimize potential contamination. During natural defecation, mid-portion stools (excluding surface layers and fecal extremes) were collected into 2 mL sterile EP tubes. Samples were immediately sealed, labeled, and transferred to a −80℃ freezer within two hours of collection to preserve microbial integrity. Rigorous Quality Control was applied, and samples lacking complete documentation or exhibiting evidence of improper storage were systematically excluded from the biobank.

Genomic DNA was initially extracted from the archived stool samples utilizing either the CTAB or SDS method. The resulting DNA purity and concentration were assessed via agarose gel electrophoresis for quality assurance. Subsequently, genomic DNA was aliquoted and diluted with sterile water to achieve a final working concentration of 1 ng/μL. PCR amplification of the target regions was then performed using this diluted genomic DNA as the template, employing barcoded specific primers, Phusion® High-Fidelity PCR Master Mix with GC Buffer (New England Biolabs), and a high-fidelity enzyme to ensure both amplification efficiency and accuracy. PCR products underwent quality validation via 2% agarose gel electrophoresis before qualified products were purified using magnetic beads and quantified via enzyme-based assays. Equimolar pooling of the final PCR products was executed based on concentration, followed by thorough mixing. The pooled library was re-checked on a 2% agarose gel, and the target bands were precisely excised and recovered using the QIAquick Gel Extraction Kit (Qiagen). Finally, libraries were constructed using the TruSeq® DNA PCR-Free Sample Preparation Kit. Library quality was quantified using Qubit and Q-PCR, and only qualified libraries were subjected to sequencing on the NovaSeq 6,000 platform.

### Microbiome data analysis

Samples were sequenced on an Illumina NovaSeq platform (LC-Bio Technologies Co., Ltd., China). Paired-end reads were assigned to samples based on their unique barcodes, merged using FLASH (v.1.2.8, USA), and quality-filtered to obtain high-quality clean tags. Chimeric sequences were filtered using the Vsearch software (v.2.3.4, China). A feature table and sequences were obtained after dereplication with DADA2 (v.1.14). Phylogenetic Analysis Taxonomic assignment of the amplicon sequence variant (ASVs) was performed using the Mothur algorithm against the SILVA SSU rRNA database (annotation threshold: min confidence 0.8). Subsequently, the community composition of each sample was statistically analyzed at the phylum, class, order, family, genus, and species levels. Rapid multiple sequence alignment was conducted using MAFFT to elucidate the phylogenetic relationships among all representative sequences.

Alpha and beta diversity indices were calculated using the R packages “phyloseq” and “vegan” after normalizing sequencing depth. To evaluate the statistical significance of community separation, Analysis of Similarities (ANOSIM) was performed based on Bray-Curtis dissimilarities using the anosim function. Subsequently, Similarity Percentage (SIMPER) analysis was employed to quantify the contribution of individual taxa to the observed inter-group dissimilarity. Differentially abundant taxa were identified using the Metastats method, which utilizes permutation-based non-parametric *t*-tests tailored for sparse microbiome data; *p*-values were adjusted for false discovery rate to obtain *q*-values. Additionally, functional metabolic profiles were predicted using PICRUSt2 based on KEGG pathway annotations.

### Statistical analysis

Statistical analysis was performed using R 4.3.3. Demographic and clinical characteristics were compared between gut frailty and non- gut frailty groups. Categorical variables were summarized as frequencies (percentages) and analyzed using the chi-square test. Normally distributed continuous variables were presented as mean ± standard deviation and compared using independent *t*-tests, while non-normally distributed continuous data were expressed as median (interquartile range) and analyzed via Mann–Whitney *U*-test. A two-sided *P* < 0.05 was considered statistically significant. Multivariable logistic regression was used to identify influencing factors in chronic heart failure patients, with a significance level set at *α* = 0.05. For alpha and beta diversity analysis, wilcoxon test were applied for two group comparison. PICRUSt2 analysis based on KEGG pathway annotation was analyzed based on Stamp differential analysis (*t*-test). Linear discriminant analysis effect size (LEfSe) was analyzed using the Kruskal–Wallis rank-sum test and the Wilcoxon rank-sum test.

## Results

### Demographic and clinical characteristics of participants

To validate the microbiome subset, the 16S rRNA sequenced participants (*n* = 60) were compared with the non-sequenced cohort (*n* = 210). No significant differences were observed across demographic, clinical, or lifestyle variables, confirming subsample representativeness (Supplementary Table S1). Within the sequenced sub-cohort, the gut frailty (*n* = 30) and non-gut frailty (*n* = 30) groups were highly comparable in age, gender, BMI, and socioeconomic background (Supplementary Table S2). Despite this demographic equivalence, the gut frailty group exhibited a significantly more severe clinical phenotype. Cardiologically, these patients demonstrated markedly impaired LVEF (*P* < 0.001) and a longer duration of CHF (*P* = 0.002). This aligned with a higher overall clinical burden, indicated by more comorbidities (*P* = 0.022) and greater polypharmacy (*P* = 0.004). Furthermore, the gut frailty group showed elevated systemic inflammation, with higher CRP (*P* = 0.007) and WBC counts (*P* = 0.017). Behaviorally and symptomatically, they also reported significantly higher PHQ-9 scores (*P* = 0.010), and prolonged sedentary time (*P* < 0.001).

Regarding the total cohort of 270 patients, comparisons stratified by gut frailty status are presented in Table 1. The gut frailty and non-gut frailty groups showed no significant differences in sex, socioeconomic factors (education, residence), or lifestyle habits such as smoking, alcohol consumption, and dietary taste preferences. Similarly, clinical parameters, including systolic blood pressure, inflammatory markers (CRP, WBC), lipid profiles (triglycerides, TC, HDL-C, LDL-C), diabetes status, and the duration of chronic heart failure, were comparable between the two groups. In contrast, significant differences were identified in age, BMI, diastolic blood pressure, and cardiac function (LVEF, NHYA stage). Furthermore, the groups differed significantly in nutritional and functional status, specifically regarding hemoglobin, total protein, albumin, MNA-HF, OFI, FP, PHQ-9, and GSRS total score, as well as in daily habits, including medication burden, sleep duration, sedentary time, exercise habits, and dietary tendencies.

**Table 1:** Comparative baseline characteristics between Gut frailty and Non-Gut frailty groups in CHF patients.

Variable	Overall	Gut Frailty	Non-Gut Frailty	*P* test
n	270	141	129	
Age (years)	69.04 (11.07)	72.82 (10.26)	64.91 (10.46)	<0.001
BMI(kg/m²)	24.35 (3.51)	23.59 (3.58)	25.18 (3.24)	<0.001
SBP (mmHg)	132.85 (22.06)	134.01 (23.33)	131.58 (20.61)	0.366
DBP (mmHg)	77.32 (13.08)	75.48 (12.66)	79.33 (13.28)	0.015
Number of medications	6.90 (2.87)	7.70 (2.99)	6.02 (2.47)	<0.001
LVEF (%)	0.59 (0.10)	0.54 (0.10)	0.63 (0.09)	<0.001
CRP (mg/L)	8.29 (18.86)	10.39 (20.18)	5.99 (17.08)	0.055
WBC (×10⁹/L)	7.10 (11.27)	6.47 (2.94)	7.78 (16.02)	0.343
Hemoglobin (g/dL)	128.42 (25.04)	122.44 (23.60)	134.95 (25.01)	<0.001
Total protein (g/L)	66.43 (6.15)	66.07 (6.30)	66.81 (5.98)	0.327
Albumin (g/L)	41.54 (4.94)	40.77 (4.55)	42.38 (5.23)	0.007
Triglycerides (mmol/L)	1.46 (1.00)	1.37 (0.66)	1.55 (1.27)	0.157
TC (mmol/L)	4.16 (1.44)	4.52 (1.42)	3.75 (1.35)	<0.001
HDL-C (mmol/L)	3.74 (1.14)	3.70 (1.16)	3.78 (1.12)	0.586
Number of comorbid chronic diseases	1.10 (0.32)	1.09 (0.34)	1.10 (0.29)	0.706
LDL-C (mmol/L)	2.13 (0.97)	2.10 (0.96)	2.15 (0.99)	0.677
FP	1.23 (1.14)	1.70 (1.16)	0.71 (0.85)	<0.001
GSRS	2.80 (2.69)	4.84 (2.11)	0.57 (0.85)	<0.001
OFI-8	2.72 (2.28)	3.59 (2.53)	1.78 (1.50)	<0.001
MNA-HF	23.64 (4.76)	21.50 (4.99)	25.98 (3.12)	<0.001
PH-9	6.29 (5.14)	8.67 (5.01)	3.70 (3.87)	<0.001
New York Heart Association(%)				<0.001
Class II	226 (83.7)	104 (73.8)	122 (94.6)	
Class III	40 (14.8)	33 (23.4)	7 ( 5.4)	
Class IV	4 ( 1.5)	4 ( 2.8)	0 ( 0.0)	
Gender (%)				0.704
Male	159 (58.9)	81 (57.4)	78 (60.5)	
Female	111 (41.1)	60 (42.6)	51 (39.5)	
Diabetes mellitus (%)				0.719
No	186 (68.9)	99 (70.2)	87 (67.4)	
Yes	84 (31.1)	42 (29.8)	42 (32.6)	
Alcohol consumption (%)				0.466
No	205 (75.9)	104 (73.8)	101 (78.3)	
Yes	65 (24.1)	37 (26.2)	28 (21.7)	
Smoking (%)				1.000
No	185 (68.5)	97 (68.8)	88 (68.2)	
Yes	85 (31.5)	44 (31.2)	41 (31.8)	
Residence (%)				0.337
Urban	171 (63.3)	85 (60.3)	86 (66.7)	
Rural	99 (36.7)	56 (39.7)	43 (33.3)	
Education level (%)				0.014
Illiterate	33 (12.2)	19 (13.5)	14 (10.9)	
Primary school	68 (25.2)	45 (31.9)	23 (17.8)	
Middle school	77 (28.5)	40 (28.4)	37 (28.7)	
High school	49 (18.1)	25 (17.7)	24 (18.6)	
Technical secondary school	8 ( 3.0)	2 ( 1.4)	6 ( 4.7)	
Associate degree	10 ( 3.7)	3 ( 2.1)	7 ( 5.4)	
University	25 ( 9.3)	7 ( 5.0)	18 (14.0)	
Sleep duration(%)				<0.001
<5 h	133 (49.3)	93 (66.0)	40 (31.0)	
>9 h	1 ( 0.4)	0 ( 0.0)	1 ( 0.8)	
6–9 h	136 (50.4)	48 (34.0)	88 (68.2)	
Sedentary time (%)				<0.001
<3 h	93 (34.4)	24 (17.0)	69 (53.5)	
>8 h	5 ( 1.9)	5 ( 3.5)	0 ( 0.0)	
3–6 h	107 (39.6)	56 (39.7)	51 (39.5)	
6–8 h	65 (24.1)	56 (39.7)	9 ( 7.0)	
Regular exercise (%)				<0.001
No	107 (39.6)	78 (55.3)	29 (22.5)	
Yes	163 (60.4)	63 (44.7)	100 (77.5)	
Chronic heart failure duration (%)				0.001
<1 year	97 (35.9)	40 (28.4)	57 (44.2)	
>5 year	83 (30.7)	57 (40.4)	26 (20.2)	
1–5 year	90 (33.3)	44 (31.2)	46 (35.7)	
Dietary preference (%)				0.003
Vegetable-rich, meat-reduced	73 (27.0)	50 (35.5)	23 (17.8)	
Meat-rich, vegetable-reduced	27 (10.0)	17 (12.1)	10 ( 7.8)	
Balanced (meat and vegetables equally)	161 (59.6)	70 (49.6)	91 (70.5)	
Strict vegetarian	9 ( 3.3)	4 ( 2.8)	5 ( 3.9)	
Dietary taste preference (%)				0.814
Bland/mild foods	215 (79.6)	111 (78.7)	104 (80.6)	
Spicy/salty foods	55 (20.4)	30 (21.3)	25 (19.4)	

Continuous variables are presented as mean ± standard deviation or median, while categorical variables are expressed as frequency (percentage).

### Univariate and multivariable logistic regression analysis of factors influencing gut frailty

To ensure statistical stability and minimize bias, variables with sparse data distributions, specifically sleep duration >9 h (*n* = 1), NYHA Class IV (*n* = 4), and sedentary time >8 h (*n* = 5), were excluded from the logistic regression models. In the univariate analysis, significant differences (*P* < 0.05) between patients with and without gut frailty were observed across three main domains: (1) demographic and clinical characteristics, including age, BMI, diastolic blood pressure, LVEF, hemoglobin, albumin, number of medications, number of comorbidities, and duration of chronic heart failure; (2) functional and psychological assessments, including the FP, OFI, MNA-HF, PHQ-9, and NYHA stage; and (3) lifestyle factors, including sleep duration, sedentary time, regular exercise habits, education level, and dietary preference. All adjusted GVIF values of these variables were less than 2, confirming the absence of significant multicollinearity (Supplementary Table S3). In the subsequent multivariable analysis adjusting for these significant confounders, dietary patterns emerged as independent predictors. A meat-rich, vegetable-reduced diet was significantly associated with a nearly 4-fold increase in the risk of gut frailty (OR = 3.995, 95% CI: 1.107–16.110, *P* = 0.041). Conversely, strict adherence to a vegetarian diet demonstrated a robust protective effect (OR = 0.148, 0.024–0.883, *P* = 0.035). Regarding other covariates, the number of comorbid chronic diseases showed a borderline association with increased risk (OR = 1.321, 1.003–1.762, *P* = 0.051), although neither reached conventional statistical significance.

### Sequencing characteristics of Gut Microbiota

**Figure 1:**
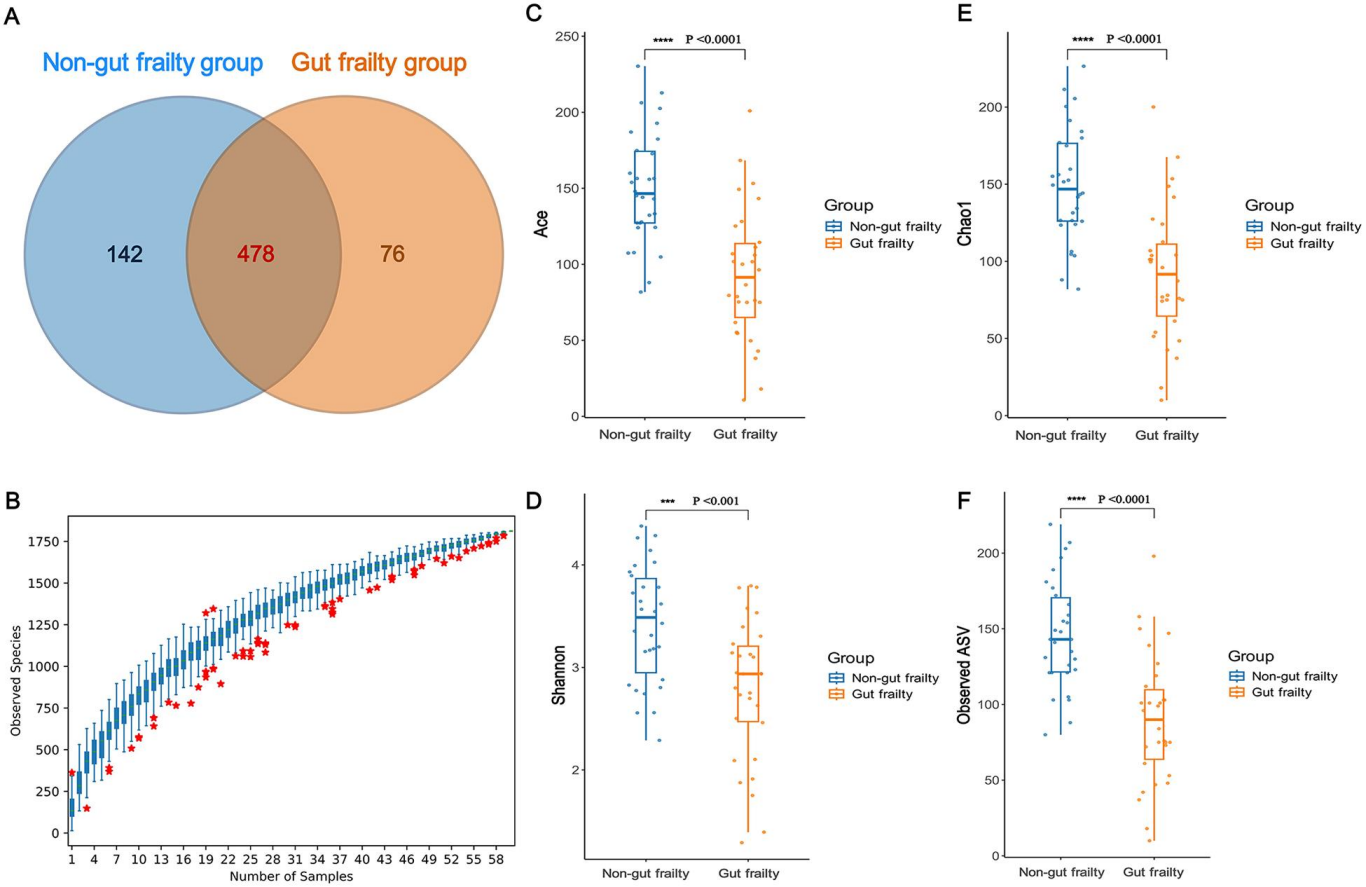
ASVs distribution, sequencing depth validation, and alpha diversity of the gut microbiota. **(A)** Venn diagram illustrating the number of shared and unique operational taxonomic units between the non-gut frailty and gut frailty groups. The overlapping region represents taxa common to both groups, while non-overlapping regions represent group-specific taxa. **(B)** Rarefaction curves based on the Observed Species index. The curves approach a plateau with increasing sequencing depth, indicating that the sampling depth was sufficient to capture the majority of microbial diversity in the samples. **(C–F)** Comparison of alpha diversity indices between the non-gut frailty and gut frailty groups. Box plots depict the **(C)** Ace index, **(D)** Shannon index, **(E)** Chao1 index, and **(F)** Observed ASV. The gut frailty group exhibited significantly lower alpha diversity across these metrics compared to the non-gut frailty group. Statistical significance is indicated by asterisks (****, *P* < 0.0001).

A total of 6,190,999 raw sequences were obtained from the 60 participant samples. Following rigorous quality control procedures, including denoising, trimming, and chimera removal, 5,312,760 high-quality sequences were retained, with sequencing depths ranging from 55,591 to 144,466 reads per sample. The species accumulation curves (Figure 1B) approached a saturation plateau, indicating that the sequencing depth was sufficient to capture the majority of microbial biodiversity within the cohort. We identified a total of 696 ASVs across the study population. As illustrated in Figure 1A, a core microbiome of 478 ASVs was shared between the two groups, reflecting a degree of compositional similarity. However, distinct group-specific features were evident: the Non-Gut Frailty group possessed a higher number of unique ASVs (*n* = 142) compared to the Gut Frailty group (*n* = 76), suggesting a loss of unique microbial taxa in the frailty condition.

**Figure 2:**
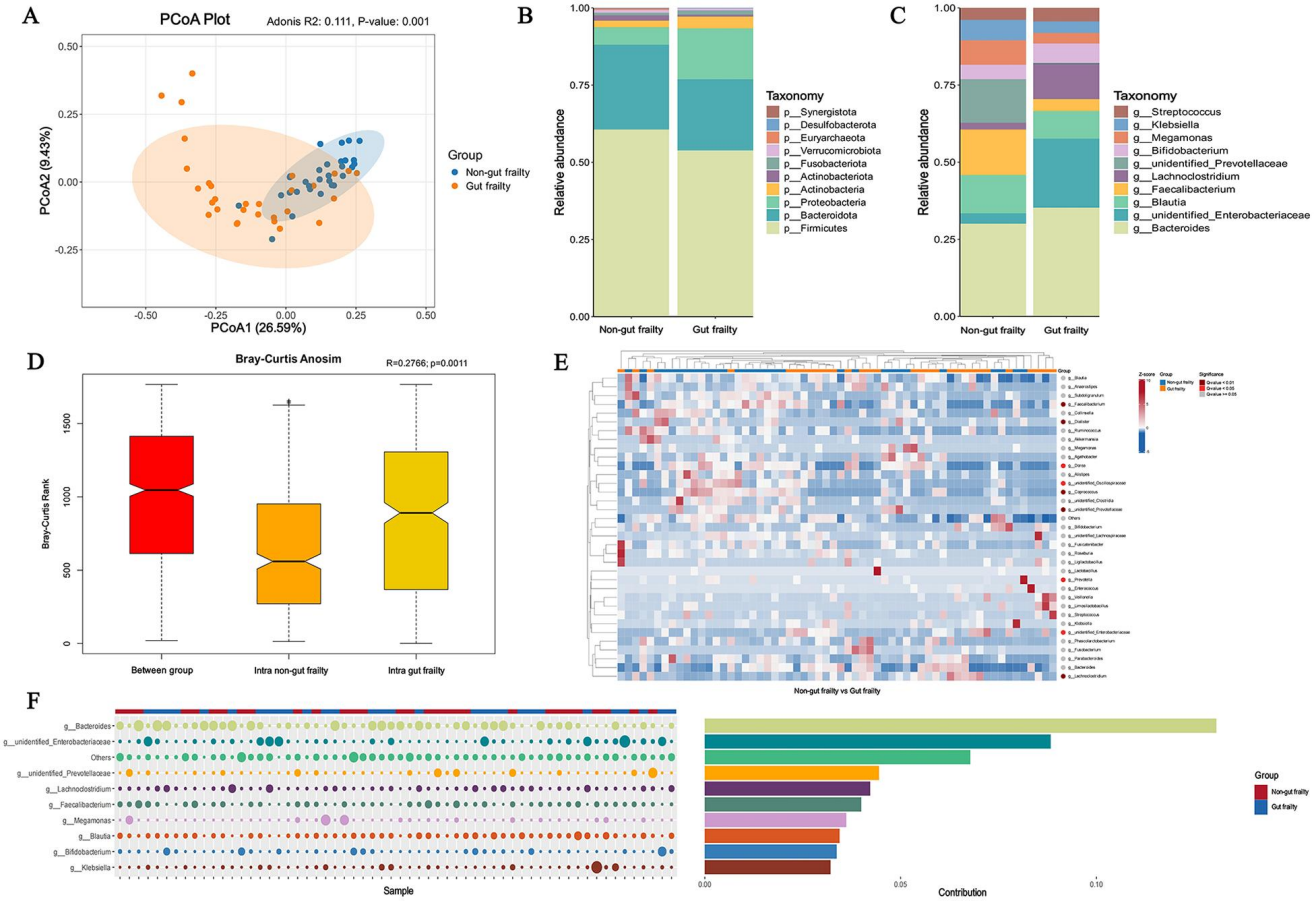
Beta diversity and taxonomic composition analysis of the gut microbiota. **(A)** Principal Coordinate Analysis (PCoA) plot based on Bray-Curtis distances, visualizing the distinct separation of microbial community structures between the non-gut frailty and gut frailty groups. The variation explained by the first two coordinates is PCoA1 (26.59%) and PCoA2 (9.43%). An Adonis test confirmed significant differences between the groups (*R*^2^ = 0.111, *P* = 0.001). **(B)** Relative abundance of dominant bacterial taxa at the Phylum level. **(C)** Relative abundance of bacterial taxa at the Genus level. **(D)** Analysis of Similarity using Bray-Curtis ranks, confirming significant dissimilarity between the groups (*R* = 0.2766, *P* = 0.0011) compared to intra-group distances. **(E)** Top 35 Genera were ranked by total quantitative abundance across all samples. The heatmap displays the Z-score standardized relative abundance, with annotations indicating statistically significant differences calculated by Metastats between the non-gut frailty and gut frailty groups. Hypothesis testing was performed to obtain *p*-values, which were corrected to q-values to assess statistical significance. **(F)** Similarity Percentage analysis identifying the top 10 specific taxa driving the compositional differences between the non-gut frailty and gut frailty groups.

Ecological diversity analyses revealed significant disparities in the gut microbiota profiles of the two groups. As depicted in Figure 2, alpha-diversity, evaluated using Observed ASVs, Shannon, Chao1, and ACE indices, differed significantly between patients with and without gut frailty. Furthermore, beta-diversity analysis was conducted to assess the extent of microbial dysbiosis. Principal Coordinates Analysis (PCoA) based on Unweighted UniFrac distances demonstrated distinct clustering patterns, with samples from the non-frail and frail groups largely occupying separate regions of the multivariate space. This structural separation was statistically confirmed by ANOSIM based on Bray-Curtis dissimilarity (*R* = 0.2766, *P* = 0.001). These results indicate that the variation in gut microbiota composition between the two groups significantly exceeds the variation within groups, confirming that gut frailty is characterized by a distinct microbial signature.

### Analysis of Gut Microbiota composition at phylum and genus levels

Taxonomic annotation at the phylum level (Figure 2B) revealed that the gut microbiota was primarily dominated by *Firmicutes* and *Bacteroidota*. Collectively, these two phyla comprised over 70% of the total relative abundance, establishing the core structural framework of the community. *Proteobacteria* and *Actinobacteriota* were consistently observed at moderate abundances, whereas low-abundance phyla, including *Verrucomicrobiota*, *Desulfobacterota*, and *Synergistota*, constituted the remaining fraction, potentially fulfilling niche-specific metabolic roles within the ecosystem. At the genus level (Figure 2C), *Bacteroides* was identified as the most prevalent taxon across the cohort. The community profile further featured a substantial presence of *Faecalibacterium*, *Blautia*, and *Unidentified_Enterobacteriaceae*, which exhibited notable variation between groups; specifically, *Faecalibacterium* appeared more abundant in the non-gut frailty group, while *Unidentified_Enterobacteriaceae* was enriched in the gut frailty group. Conversely, other genera such as *Streptococcus*, *Klebsiella*, *Megamonas*, and *Bifidobacterium* were detected at relatively lower abundances, contributing to the diversity of the secondary community cluster.

### Differential analysis of the Gut microbiome composition

To identify specific microbial signatures associated with gut frailty, we performed a differential abundance analysis using the Metastats method. This screening identified 39 genus-level taxa that differed significantly between the groups (*p*-value < 0.05; Supplementary Table S4). Among these, 8 genera were enriched in the gut frailty group, while 31 genera were depleted. To further elucidate the structural alterations associated with this phenotype, we prioritized the top 35 genera ranked by total quantitative abundance across all samples and visualized their distribution via a heatmap integrated with statistical significance (Figure 2E). This analysis highlighted that the separation between phenotypes was partly associated with significant shifts in several dominant taxa (*q*-value < 0.05). Specifically, beneficial producers such as *Faecalibacterium*, *Dialister*, *Coprococcus*, and *unidentified Prevotellaceae* were significantly depleted in the gut frailty group, whereas *Lachnoclostridium* was significantly enriched. Collectively, these marked fluctuations in abundance-ranked genera indicate that the gut frailty phenotype in heart failure patients is characterized by a distinct state of dysbiosis.

**Figure 3:**
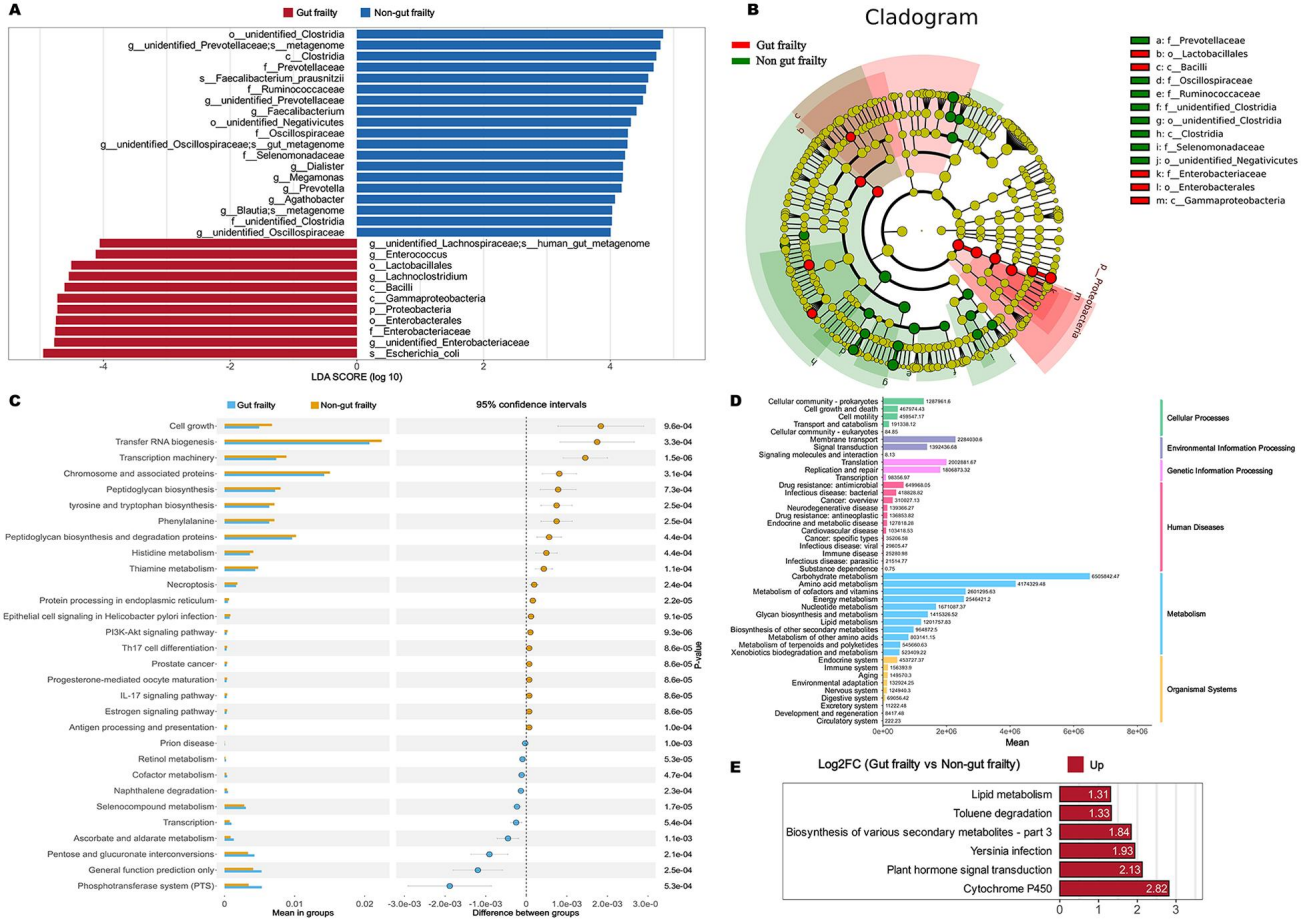
Identification of characteristic taxa and predicted functional pathways of the gut microbiota. **(A,B)** Linear discriminant analysis Effect Size (LEfSe) analysis identifying differentially abundant taxa biomarkers between the non-gut frailty and gut frailty groups. **(A)** Histogram of Linear Discriminant Analysis scores. Taxa enriched in the gut frailty group are shown with positive scores, while taxa enriched in the non-gut frailty group are shown with negative scores. **(B)** Cladogram illustrating the phylogenetic relationship and taxonomic hierarchy of the identified differential taxa, from phylum to species levels. **(C)** Prediction of functional potential using PICRUSt2 analysis. The extended error bar plot displays significantly different KEGG pathways at Level 3 between the two groups. Statistical significance was determined using a *T*-test, showing the mean proportion and 95% confidence intervals of the difference. **(D)** Heatmap of predicted functional profiles based on KEGG pathways, visualizing the differential enrichment of functional categories across the groups. **(E)** Differential abundance analysis of metabolic pathways performed using the metagenomeSeq method. The bar plot illustrates the Log_2_ Fold Change of pathways, highlighting those significantly upregulated or downregulated in the gut frailty group compared to the non-gut frailty group.

Furthermore, we employed a SIMPER analysis utilizing the Bray-Curtis dissimilarity metric to dissect the specific taxonomic contributors to the structural shifts observed between the non-gut frailty and gut frailty group. This approach isolated the top 10 genera primarily driving the compositional divergence between the two cohorts. As depicted in Figure 2F, these key taxa are ranked by their contribution to the total dissimilarity, with bubble magnitude reflecting their relative abundance. Notably, *Bacteroides* and *Faecalibacterium* emerged as the primary drivers of community variation. Moreover, the analysis underscored substantial contributions from *Blautia*, *Megamonas*, and *Bifidobacterium*, alongside potential pathobionts such as *Klebsiella* and *Lachnoclostridium*. Moreover, an LEfSe was conducted to screen for microbiota with significant differences in abundance between the two groups to identify the appropriate biomarkers. As depicted in Figure 3B, the microbial species labeled red were enriched in the gut frailty group, whereas those labeled green were enriched in control group. Potential biomarkers with linear discriminant analysis values >4 are shown in Figure 3A.

### Potential functional prediction analysis

To elucidate the functional implications of the observed taxonomic shifts, PICRUSt2 was utilized to predict the metabolic potential of the gut microbiota. At a broad level, the functional landscape of both groups was dominated by Metabolism, which constituted the most abundant Level 1 category, primarily correlated with carbohydrate and amino acid metabolism pathways (Figure 3D). However, differential analysis (level 3T-test; Figure 3C) revealed distinct functional signatures characterizing the gut frailty phenotype. While the non-gut frailty group was enriched in pathways essential for cell growth and genetic information processing, specifically transfer RNA biogenesis, transcription machinery, and peptidoglycan biosynthesis, the gut frailty group exhibited a marked shift toward xenobiotic biodegradation and environmental adaptation. Notably, the gut frailty group showed significant upregulation of Cytochrome P450 (log_2_ FC = 2.82), toluene degradation, and lipid metabolism (Figure 3E). Furthermore, differential analysis confirmed significant enrichments in the phosphotransferase system and pentose and glucuronate interconversions in the frailty group (*P* < 0.05). Collectively, these results suggest a functional transition from core replication and biosynthesis in the non-frail state to a stress-responsive profile in gut frailty, characterized by enhanced xenobiotic metabolism and altered carbohydrate transport mechanisms.

## Discussion

To our knowledge, this is one of the first studies to comprehensively characterize the concept of “gut frailty” in patients with CHF by integrating clinical phenotypes with 16S rRNA-based microbiome profiling. Two principal findings emerged from our analysis. First, gut frailty appears to represent a distinct clinical sub-phenotype in CHF that is closely associated with dietary pattern: high meat consumption was linked to increased susceptibility, while plant-based dietary habits showed a protective association. Second, the gut microbiota of patients with gut frailty exhibited a functional shift from a regenerative, saccharolytic profile toward a stress-responsive, proteolytic configuration marked by enrichment in xenobiotic metabolism pathways.

Our analysis underscored the substantial association between diet and gut frailty. A meat-rich, vegetable-reduced dietary pattern was associated with a nearly four-fold increase in the odds of gut frailty, whereas strict vegetarianism was associated with markedly lower odds. These observations are broadly consistent with the “gut hypothesis” of heart failure. High intake of red meat provides abundant substrates for microbial production of trimethylamine (TMA) (14), which undergoes hepatic oxidation to trimethylamine *N*-oxide (TMAO), a metabolite increasingly recognized for its pro-atherogenic and pro-fibrotic properties (15, 16). Several prospective cohort studies have reported that elevated plasma TMAO concentrations are associated with adverse outcomes in heart failure, including higher mortality (17, 18). Notably, our microbiome analysis revealed a significant enrichment of *Lachnoclostridium* in the gut frailty group. Members of these genus are established TMA-producers (19). The co-occurrence of a meat-rich diet as a clinical risk factor and the enrichment of TMA-producing bacteria suggests that gut frailty may, at least in part, reflect the gastrointestinal manifestation of a diet-microbiota-TMAO axis that contributes to systemic deterioration in CHF.

Conversely, the protective association of a vegetarian diet is likely underpinned by the high intake of non-digestible carbohydrates and dietary fiber. These substrates preferentially support saccharolytic fermentation by genera such as *Faecalibacterium*, which was enriched in our non-frailty group, and promote the colonic production of SCFAs, particularly butyrate (20, 21). SCFAs contribute to gut homeostasis through at least two complementary mechanisms. At the mucosal level, butyrate serves as the principal oxidative fuel for colonocytes and has been shown to upregulate tight junction proteins including ZO-1 and occludin (22), thereby reinforcing epithelial barrier integrity and limiting translocation of luminal endotoxins into the systemic circulation. Once translocated, LPS activates TLR4 signaling on immune cells and cardiomyocytes, triggering a cascade of pro-inflammatory cytokines such as TNF-α and IL-6 that directly suppress myocardial contractility and promote cardiac remodeling (2). This endotoxin-driven inflammatory loop, termed metabolic endotoxemia, is now widely regarded as a central pathophysiological link between intestinal barrier dysfunction and the progression of heart failure (23). At the systemic level, SCFAs exert additional anti-inflammatory effects through inhibition of histone deacetylases and activation of G-protein-coupled receptors, attenuating pro-inflammatory cytokine signaling (24). In this context, the marked depletion of *Faecalibacterium* observed in the gut frailty is noteworthy, as it reflects the loss of a key butyrate-producing taxon with established cardioprotective relevance. Recent evidence suggests that butyrate derived from *Faecalibacterium prausnitzii* may protect against age-related cardiac dysfunction by suppressing cardiomyocyte ferroptosis (20), further highlighting the functional significance of this taxon within the gut-heart axis.

Ecologically, the gut frailty phenotype in patients with CHF was characterized by a significant reduction in alpha-diversity and distinct compositional separation in beta-diversity, indicating an erosion of microbial resilience that may predispose the ecosystem to opportunistic colonization (25, 26). At the phylum level, a disproportionate expansion of *Proteobacteria* was observed, a finding of particular clinical relevance given that *Proteobacteria* enrichment has been proposed as a microbial signature of ecosystem instability and host disease (27). At a finer taxonomic resolution, the enrichment of *Enterobacteriaceae* and *Klebsiella* within the gut frailty group offers mechanistic insight into the perpetuation of this dysbiosis. As facultative anaerobes, these pathobionts thrive under conditions of epithelial oxygen leakage (28). In advanced CHF, chronic splanchnic ischemia–reperfusion injury and mucosal edema generate reactive oxygen species in the gut lumen, thereby disrupting the physiologically anaerobic milieu and conferring a selective growth advantage to *Proteobacteria* over obligate anaerobes. The resultant overgrowth of these gram-negative taxa substantially amplifies the luminal LPS burden. While metabolic endotoxemia has been implicated in the pathogenesis of heart failure, our data suggest that the gut frailty phenotype may represent a critical inflection point at which endotoxin translocation transitions from a compensated, low-grade process to a decompensated, high-flux state (2, 29). The convergence of an expanded endotoxin reservoir with a severely compromised intestinal barrier would be expected to sustain a level of systemic inflammatory activation.

Our PICRUSt2-based functional prediction analysis revealed notable metabolic reprogramming associated with gut frailty. The microbiome of non-frail patients was functionally oriented toward growth and repair, which is consistent with a resilient, homeostatic ecosystem that actively supports mucosal barrier maintenance. In contrast, the gut frailty microbiome exhibited a stress-response phenotype dominated by xenobiotic biodegradation, cytochrome P450 activity, and toluene degradation pathways. Emerging pharmacomicrobiomics evidence indicates that gut bacteria possess that capacity to metabolize over 176 commonly used drugs (30). The observed upregulation of bacterial cytochrome P450 pathways may thus represent the microbiome's metabolic response to the high burden of exogenous pharmaceutical stressors, potentially altering drug bioavailability or generating toxic intermediates (30). Furthermore, the enrichment of toluene degradation pathways may indicate an accumulation of environmental toxins due to impaired intestinal motility and clearance. However, it should also be acknowledged that the functional profiles reported here were computationally predicted from 16S rRNA amplicon data using PICRUSt2 rather than directly measured. While this approach offers valuable hypothesis-generating insights, it cannot capture strain-level functional variation or post-transcriptional regulation, and its accuracy depends on the completeness of reference genome databases. The functional shifts we describe should therefore be regarded as indicative of metabolic potential rather than confirmed biochemical activity, and validation through shotgun metagenomics and targeted metabolomics will be necessary to confirm these predictions and establish their clinical relevance.

Several potential confounders also warrant consideration. Patients with CHF typically receive polypharmacy regimens encompassing statins, beta-blockers, diuretics, renin–angiotensin system inhibitors, and proton pump inhibitors, many of which independently reshape gut microbial composition and metabolic output (31, 32). Proton pump inhibitors, for example, elevate gastric pH and thereby promote *Enterobacteriaceae* overgrowth (33), whereas metformin, frequently co-prescribed for comorbid diabetes, selectively enriches *Escherichia* and *Akkermansia (*34). Our multivariable regression model did account for medication burden and chronic comorbidities, yet we did not undertake drug-class-level stratification within the microbiome sub-cohort, leaving open the question of how individual agents may have contributed to the taxonomic shifts we report. Antibiotic exposure poses a parallel challenge. Although patients with recent antibiotic use were excluded at enrollment, the lingering ecological effects of earlier courses, including selective depletion of obligate anaerobes and facilitation of resistant *Proteobacteria* blooms, are difficult to quantify and may partly underlie the *Proteobacteria* enrichment seen in the frailty group (35). Compounding these pharmacological influences, the heavy comorbidity burden characteristic of elderly CHF patients, such as diabetes mellitus, chronic kidney disease, and chronic obstructive pulmonary disease, can itself perturb microbial communities through intersecting metabolic, immunological, and drug-related pathways. Fully disentangling these overlapping influences from intrinsic gut–heart axis pathophysiology will require future studies incorporating detailed drug-class stratification, comprehensive antibiotic-exposure histories, and integrated multi-omics profiling.

Importantly, the observed dysbiosis in our cohort must be interpreted against the advanced age of the study population, as aging itself drives substantial remodeling of the gut microbiome, characterized by reduced alpha-diversity, loss of beneficial *Bifidobacterium* and *Faecalibacterium*, and a proportional expansion of pro-inflammatory *Proteobacteria (*36). That said, the two groups in our study were well matched for age, which argues against aging alone as the primary driver of the microbial differences we observed; rather, these signatures appear more closely tied to disease-related mechanisms. Even so, one cannot ignore the possibility that CHF-specific insults act upon an already age-remodeled microbial landscape to synergistically hasten the progression toward gut frailty. Future investigations employing age-stratified designs or incorporating healthy age-matched controls will be essential to tease apart the independent and interactive contributions of aging and heart failure to intestinal dysbiosis.

Several limitations should be acknowledged. First, the cross-sectional design prevents causal inference; we cannot definitively determine whether dysbiosis drives gut frailty or develops as a consequence of it. Second, although the representativeness of our sequencing sub-cohort (*n* = 60) was confirmed through rigorous statistical comparisons, the relatively small sample size necessitates validation in larger, multi-center cohorts. Third, while we adjusted for the total burden of medications and comorbidities in our clinical models, fully disentangling their direct ecological impact on the gut microbiota remains methodologically challenging. Fourth, as 16S rRNA sequencing provides predictive functional data, future targeted metabolomics and detailed drug-class stratification are critically needed to definitively uncouple the intrinsic pathophysiology of the gut-heart axis from CHF pharmacological artifacts. Fifth, our current classification of dietary patterns is qualitative. Subsequent investigations may employ robust quantitative instruments, such as standardized Food Frequency Questionnaires or 24 h dietary recalls. To transcend these observational limitations, future research must decisively transition toward targeted intervention studies. Specifically, randomized controlled trials are critically warranted to evaluate whether structured high-fiber dietary interventions—or microbiome-directed therapies such as *Faecalibacterium*-based next-generation probiotics—can effectively restore mucosal barrier integrity, attenuate systemic endotoxemia, and alleviate gastrointestinal symptoms. Ultimately, executing these interventional paradigms will establish whether modulating the gut-heart axis can be leveraged as a disease-modifying strategy to reverse systemic frailty in advanced heart failure.

## Data Availability

The raw sequencing data supporting the findings of this study have been deposited in the NCBI Sequence Read Archive (SRA) under the BioProject accession number PRJNA1284067. These data are publicly available and can be accessed through the NCBI SRA database.

## References

[1] Tanai E Frantz S. Pathophysiology of heart failure. Compr Physiol. (2016) 6(1):187–214. 10.1002/j.2040-4603.2016.tb00669.x 26756631

[2] Tang WHW Li DY Hazen SL. Dietary metabolism, the gut microbiome, and heart failure. Nat Rev Cardiol. (2019) 16(3):137–54. 10.1038/s41569-018-0108-7 30410105 PMC6377322

[3] Sandek A Swidsinski A Schroedl W Watson A Valentova M Herrmann R. Intestinal blood flow in patients with chronic heart failure: a link with bacterial growth, gastrointestinal symptoms, and cachexia. J Am Coll Cardiol. (2014) 64(11):1092–102. 10.1016/j.jacc.2014.06.1179 25212642

[4] Romeiro FG Okoshi K Zornoff LA Okoshi MP. Gastrointestinal changes associated to heart failure. Arq Bras Cardiol. (2012) 98(3):273–7. 10.1590/S0066-782X2012000300011 22527026

[5] Sunayama T Matsue Y Dotare T Maeda D Iso T Morisawa T. Multidomain frailty as a therapeutic target in elderly patients with heart failure. Int Heart J. (2022) 63(1):1–7. 10.1536/ihj.21-839 35095060

[6] Rémond D Shahar DR Gille D Pinto P Kachal J Peyron MA. Understanding the gastrointestinal tract of the elderly to develop dietary solutions that prevent malnutrition. Oncotarget. (2015) 6(16):13858–98. 10.18632/oncotarget.4030 26091351 PMC4546438

[7] Sundaram V Fang JC. Gastrointestinal and liver issues in heart failure. Circulation. (2016) 133(17):1696–703. 10.1161/CIRCULATIONAHA.115.020894 27143152

[8] Naito Y. Gut frailty: its concept and pathogenesis. Digestion. (2024) 105(1):49–57. 10.1159/000534733 37967548 PMC10777716

[9] Chahal-Kummen M Blom-Høgestøl IK Eribe I Klungsøyr O Kristinsson J Mala T. Abdominal pain and symptoms before and after Roux-en-Y gastric bypass. BJS Open. (2019) 3(3):317–26. 10.1002/bjs5.50148 31183448 PMC6551394

[10] Dwairi R Al-Refu K Aldiabat B Al-Smirat H Alnawaiseh NA Alhalabi W. Modality of dialysis and gastrointestinal symptoms: a cross-sectional study in Jordanian adults. Kidney Dial. (2026) 6(1):1. 10.3390/kidneydial6010001

[11] Tanabe H Higurashi T Takatsu T Misawa N Yoshihara T Goto S. Effects of colorectal endoscopic submucosal dissection on postoperative abdominal symptoms: a prospective observational study. Surg Endosc. (2022) 36(1):314–20. 10.1007/s00464-020-08278-w 33502617

[12] Pan Y Yang J Jiang Y Feng ZQ Tang XY Yuan Y. Gut frailty: the core of reversing frailty. Ann Med. (2025) 57(1):2527955. 10.1080/07853890.2025.2527955 40616646 PMC12231257

[13] Kulich KR Madisch A Pacini F Piqué JM Regula J Van Rensburg CJ. Reliability and validity of the gastrointestinal symptom rating scale (GSRS) and quality of life in reflux and dyspepsia (QOLRAD) questionnaire in dyspepsia: a six-country study. Health Qual Life Outcomes. (2008) 6:12. 10.1186/1477-7525-6-12 18237386 PMC2276197

[14] Yoshida E Arage G Rašo LM Merino J Larsson SC Ahmad S. Meat intake in relation to metabolomic signatures, gut microbiome, and cardiovascular disease risk: a narrative review. Nutr Rev. (2025). 10.1093/nutrit/nuaf234 41370089

[15] Lee Y Nemet I Wang Z Lai HTM de Oliveira Otto MC Lemaitre RN. Longitudinal plasma measures of trimethylamine N-oxide and risk of atherosclerotic cardiovascular disease events in community-based older adults. J Am Heart Assoc. (2021) 10(17):e020646. 10.1161/JAHA.120.020646 34398665 PMC8649305

[16] Wang Z Klipfell E Bennett BJ Koeth R Levison BS DuGar B. Gut flora metabolism of phosphatidylcholine promotes cardiovascular disease. Nature. (2011) 472(7341):57–63. 10.1038/nature09922 21475195 PMC3086762

[17] Wang M Li XS Wang Z de Oliveira Otto MC Lemaitre RN Fretts A. Trimethylamine N-oxide is associated with long-term mortality risk: the multi-ethnic study of atherosclerosis. Eur Heart J. (2023) 44(18):1608–18. 10.1093/eurheartj/ehad089 36883587 PMC10411925

[18] Lu X Liu J Zhou B Wang S Liu Z Mei F. Microbial metabolites and heart failure: friends or enemies? Front Microbiol. (2022) 13:956516. 10.3389/fmicb.2022.956516 36046023 PMC9420987

[19] Cai YY Huang FQ Lao X Lu Y Gao X Alolga RN. Integrated metagenomics identifies a crucial role for trimethylamine-producing lachnoclostridium in promoting atherosclerosis. NPJ Biofilms Microbiomes. (2022) 8(1):11. 10.1038/s41522-022-00273-4 35273169 PMC8913745

[20] Zhang Y Wei Y Han X Shi L Yu H Ji X. Faecalibacterium prausnitzii prevents age-related heart failure by suppressing ferroptosis in cardiomyocytes through butyrate-mediated LCN2 regulation. Gut Microbes. (2025) 17(1):2505119. 10.1080/19490976.2025.2505119 40364435 PMC12080280

[21] Cronin P Joyce SA O'Toole PW O'Connor EM. Dietary fibre modulates the gut Microbiota. Nutrients. (2021) 13(5):1655. 10.3390/nu13051655 34068353 PMC8153313

[22] Parada Venegas D De la Fuente MK Landskron G González MJ Quera R Dijkstra G. Short chain fatty acids (SCFAs)-mediated gut epithelial and immune regulation and its relevance for inflammatory bowel diseases. Front Immunol. (2019) 10:277. 10.3389/fimmu.2019.00277 30915065 PMC6421268

[23] Chulenbayeva L Issilbayeva A Sailybayeva A Bekbossynova M Kozhakhmetov S Kushugulova A. Short-Chain fatty acids and their metabolic interactions in heart failure. Biomedicines. (2025) 13(2):343. 10.3390/biomedicines13020343 40002756 PMC11853371

[24] Li M van Esch B Wagenaar GTM Garssen J Folkerts G Henricks PAJ. Pro- and anti-inflammatory effects of short chain fatty acids on immune and endothelial cells. Eur J Pharmacol. (2018) 831:52–9. 10.1016/j.ejphar.2018.05.003 29750914

[25] Lozupone CA Stombaugh JI Gordon JI Jansson JK Knight R. Diversity, stability and resilience of the human gut microbiota. Nature. (2012) 489(7415):220–30. 10.1038/nature11550 22972295 PMC3577372

[26] Báez-Ferrer N Lemus-Martín A Castro-Hernández MB Avanzas P Martínez-González S Lecuona-Fernández M. Gut Microbiota alterations in heart failure patients: insights from a systematic review. J Clin Med. (2025) 14(22):8110. 10.3390/jcm14228110 41303145 PMC12653670

[27] Shin NR Whon TW Bae JW. Proteobacteria: microbial signature of dysbiosis in gut microbiota. Trends Biotechnol. (2015) 33(9):496–503. 10.1016/j.tibtech.2015.06.011 26210164

[28] Zeng MY Inohara N Nuñez G. Mechanisms of inflammation-driven bacterial dysbiosis in the gut. Mucosal Immunol. (2017) 10(1):18–26. 10.1038/mi.2016.75 27554295 PMC5788567

[29] Cani PD Amar J Iglesias MA Poggi M Knauf C Bastelica D. Metabolic endotoxemia initiates obesity and insulin resistance. Diabetes. (2007) 56(7):1761–72. 10.2337/db06-1491 17456850

[30] Zimmermann M Zimmermann-Kogadeeva M Wegmann R Goodman AL. Mapping human microbiome drug metabolism by gut bacteria and their genes. Nature. (2019) 570(7762):462–7. 10.1038/s41586-019-1291-3 31158845 PMC6597290

[31] Nagata N Nishijima S Miyoshi-Akiyama T Kojima Y Kimura M Aoki R. Population-level metagenomics uncovers distinct effects of multiple medications on the human gut microbiome. Gastroenterology. (2022) 163(4):1038–52. 10.1053/j.gastro.2022.06.070 35788347

[32] Vich Vila A Collij V Sanna S Sinha T Imhann F Bourgonje AR. Impact of commonly used drugs on the composition and metabolic function of the gut microbiota. Nat Commun. (2020) 11(1):362. 10.1038/s41467-019-14177-z 31953381 PMC6969170

[33] Zhang J Zhang C Zhang Q Yu L Chen W Xue Y. Meta-analysis of the effects of proton pump inhibitors on the human gut microbiota. Bmc Microbiol. (2023) 23(1):171. 10.1186/s12866-023-02895-w 37337143 PMC10278323

[34] Rosell-Díaz M Fernández-Real JM. Metformin, cognitive function, and changes in the gut microbiome. Endocr Rev. (2024) 45(2):210–26. 10.1210/endrev/bnad029 37603460 PMC10911951

[35] Fishbein SRS Mahmud B Dantas G. Antibiotic perturbations to the gut microbiome. Nat Rev Microbiol. (2023) 21(12):772–88. 10.1038/s41579-023-00933-y 37491458 PMC12087466

[36] Shen Y Fan N Ma SX Cheng X Yang X Wang G. Gut Microbiota dysbiosis: pathogenesis, diseases, prevention, and therapy. MedComm. (2025) 6(5):e70168. 10.1002/mco2.70168 40255918 PMC12006732

