# Supplementary material for: Gut frailty in chronic heart failure: clinical determinants and distinct microbial signatures
Source: Front Cardiovasc Med. 2026 Jun 19;13:1779873. doi: 10.3389/fcvm.2026.1779873 (PMC13328328; doi:10.3389/fcvm.2026.1779873)
Supplement: Supplementary file 1 [file Datasheet1.docx]

**Supplementary Method**

**1. The inclusion and exclusion criteria**

The inclusion criteria included:

(1) Diagnosis of CHF meeting the 2024 Chinese Guidelines for the Diagnosis and Treatment of Heart Failure, with clinically stable status; (2) Intact cognitive function and full compliance with study protocols; (3) Informed consent provided; (4) New York Heart Association (NYHA) functional class II-IV; (5) Age ≥18 years; (6) Newly hospitalized patients (≤24 h post-admission).

The exclusion criteria included:

(1) Severe cardiovascular conditions, including arrhythmia, myocardial infarction, or end-stage CHF*; (2) Uncontrolled chronic diseases, such as valvular heart disease, malignancy, autoimmune disorders, unstable hepatic/renal/pulmonary diseases, severe GI disorders, or acute complications; (3) Oral pathologies impairing food intake; (4) Concurrent participation in interventional trials; (5) Dietary restrictions for specific intervention, including colonoscopy or fecal occult blood tests; (6) Refusal to provide stool samples post-consent; (7) Chronic diarrhea or acute gastroenteritis within 1 week; (8) Probiotic use within 4 weeks; (9) Unbalanced diet, including spicy/raw/contaminated foods, within 1 week.

* The detailed definition of severe cardiovascular conditions:

(1) Diagnosis of end-stage CHF required:

A. Severe Symptom Burden: severe or persistent symptoms of heart failure, corresponding to NYHA functional class III or IV;

B. Severe Cardiac Dysfunction: objective evidence of severe dysfunction, defined as meeting at least one of the following conditions:

1. Severe left ventricular systolic dysfunction (Left Ventricular Ejection Fraction [LVEF]≤30%);

2. Isolated right ventricular failure;

3. Inoperable severe valvular heart disease or complex congenital anomalies;

4. Persistently elevated Brain Natriuretic Peptide (BNP) or N-terminal pro-BNP (NT-proBNP) levels in the context of documented significant diastolic dysfunction.

C. Recurrent hospitalizations: a history of recurrent heart failure decompensation within the preceding 12 months (defined as ≥1 unplanned hospitalization or emergency department visit) for events requiring:

1. High-dose IV diuretics;

2. Low cardiac output requiring inotropes or vasoactive drugs;

3. Malignant arrhythmias.

D. Severely impaired exercise capacity: objective evidence of severe functional impairment, defined by at least one of the following:

1. 6-min walk distance (6MWD) <300 meters;

2. Peak oxygen uptake (peak VO₂) <12 mL/kg/min or <50% of the predicted value during cardiopulmonary exercise testing.

(2) Refractory end-stage CHF required exclusion of reversible causes and maximized therapy.

**2. Sample size estimation**

Initially, a pilot study involving 35 participants was conducted to estimate the required sample size, which estimated a gut frailty prevalence (p) of 60%. Based on this prevalence, and using a two-tailed significance level (α) of 0.05 and a margin of error (d) of 0.06, the calculated required sample size was 256. Accounting for a 10% anticipated rate of invalid or non-response, the final target for recruitment was 281 patients. Ultimately, 11 participants were lost to follow-up, and a total of 270 patients were enrolled for further analysis.

**Table S1. The baseline of demographic, clinical and lifestyle factors between 16S rRNA and non-16S rRNA groups**.

| **Variable** | **NO 16SrRNA** | **16SrRNA** | **P test** | **SMD** |
| --- | --- | --- | --- | --- |
| n | 210 | 60 |  |  |
| Age (years) | 69.55 (11.30) | 67.25 (10.12) | 0.156 | 0.215 |
| BMI (kg/m²) | 24.43 (3.52) | 24.06 (3.47) | 0.474 | 0.105 |
| SBP (mmHg) | 134.17 (22.34) | 128.23 (20.58) | 0.066 | 0.276 |
| DBP (mmHg) | 77.81 (13.35) | 75.60 (12.03) | 0.249 | 0.174 |
| Number of medications | 7.02 (3.07) | 6.45 (2.01) | 0.173 | 0.221 |
| LVEF (%) | 0.59 (0.10) | 0.57 (0.11) | 0.169 | 0.199 |
| CRP (mg/L) | 8.45 (20.36) | 7.71 (12.35) | 0.788 | 0.044 |
| WBC (×10⁹/L) | 7.35 (12.74) | 6.20 (1.83) | 0.486 | 0.127 |
| Hemoglobin (g/dL) | 128.32 (25.73) | 128.77 (22.63) | 0.903 | 0.018 |
| Total protein (g/L) | 66.26 (6.09) | 66.99 (6.39) | 0.419 | 0.117 |
| Albumin (g/L) | 41.44 (4.45) | 41.86 (6.41) | 0.57 | 0.075 |
| Triglycerides (mmol/L) | 1.40 (0.76) | 1.65 (1.58) | 0.091 | 0.200 |
| TC (mmol/L) | 3.74 (1.15) | 3.75 (1.13) | 0.944 | 0.010 |
| HDL-C (mmol/L) | 1.11 (0.30) | 1.06 (0.38) | 0.317 | 0.136 |
| Number of comorbid chronic diseases | 4.19 (1.53) | 4.03 (1.06) | 0.456 | 0.120 |
| LDL-C (mmol/L) | 2.15 (0.98) | 2.06 (0.95) | 0.534 | 0.092 |
| Frailty Phenotype (FP) | 1.25 (1.20) | 1.15 (0.90) | 0.558 | 0.092 |
| Gastrointestinal Symptom Rating Scale (GSRS) | 2.79 (2.64) | 2.83 (2.87) | 0.913 | 0.016 |
| Oral Frailty Index-8 (OFI-8) | 2.74 (2.30) | 2.67 (2.26) | 0.831 | 0.031 |
| Mini Nutritional Assessment-Heart Failure (MNA-HF) | 23.52 (4.96) | 24.08 (3.97) | 0.424 | 0.124 |
| Patient Health Questionnaire-9 (PHQ-9) | 6.53 (5.49) | 5.45 (3.54) | 0.15 | 0.235 |
| New York Heart Association (%) |  |  | 0.99 | 0.021 |
| Class II | 176 (83.8) | 50 (83.3) |  |  |
| Class III | 31 (14.8) | 9 (15.0) |  |  |
| Class IV | 3 (1.4) | 1 (1.7) |  |  |
| Gender = Female (%) | 85 (40.5) | 26 (43.3) | 0.804 | 0.058 |
| Diabetes mellitus = yes (%) | 61 (29.0) | 23 (38.3) | 0.225 | 0.197 |
| Alcohol consumption = yes (%) | 50 (23.8) | 15 (25.0) | 0.985 | 0.028 |
| Smoking = yes (%) | 62 (29.5) | 23 (38.3) | 0.255 | 0.187 |
| Residence = rural (%) | 74 (35.2) | 25 (41.7) | 0.448 | 0.132 |
| Education level (%) |  |  | 0.987 | 0.143 |
| Illiterate | 25 (11.9) | 8 (13.3) |  |  |
| Primary school | 55 (26.2) | 13 (21.7) |  |  |
| Middle school | 59 (28.1) | 18 (30.0) |  |  |
| High school | 38 (18.1) | 11 (18.3) |  |  |
| Technical secondary school | 6 (2.9) | 2 (3.3) |  |  |
| Associate degree | 7 (3.3) | 3 (5.0) |  |  |
| University | 20 (9.5) | 5 (8.3) |  |  |
| Sleep duration (%) |  |  | 0.85 | 0.102 |
| ＜5h | 104 (49.5) | 29 (48.3) |  |  |
| ＞9h | 1 (0.5) | 0 (0.0) |  |  |
| 6-9h | 105 (50.0) | 31 (51.7) |  |  |
| Sedentary time (%) |  |  | 0.582 | 0.244 |
| ＜3h | 70 (33.3) | 23 (38.3) |  |  |
| ＞8h | 5 (2.4) | 0 (0.0) |  |  |
| 3-6h | 83 (39.5) | 24 (40.0) |  |  |
| 6-8h | 52 (24.8) | 13 (21.7) |  |  |
| Regular exercise = yes (%) | 124 (59.0) | 39 (65.0) | 0.495 | 0.123 |
| Chronic heart failure duration (%) |  |  | 0.647 | 0.139 |
| ＜1 | 74 (35.2) | 23 (38.3) |  |  |
| ＞5 | 63 (30.0) | 20 (33.3) |  |  |
| 1-5 | 73 (34.8) | 17 (28.3) |  |  |
| Dietary preference (%) |  |  | 0.475 | 0.236 |
| Vegetable-rich, meat-reduced | 53 (25.2) | 20 (33.3) |  |  |
| Meat-rich, vegetable-reduced | 20 (9.5) | 7 (11.7) |  |  |
| Balanced (meat and vegetables equally) | 129 (61.4) | 32 (53.3) |  |  |
| Strict vegetarian | 8 (3.8) | 1 (1.7) |  |  |
| Dietary taste preference = spicy/salty foods (%) | 44 (21.0) | 11 (18.3) | 0.793 | 0.066 |

BMI: Body Mass Index; SBP: Systolic Blood Pressure; DBP: Diastolic Blood Pressure; LVEF: Left Ventricular Ejection Fraction; CRP: C-Reactive Protein; WBC: White Blood Count; TC: Total Cholesterol; HDL-C: High-Density Lipoprotein Cholesterol; LDL-C: Low-Density Lipoprotein Cholesterol; FP: Frailty Phenotype; GSRS: Gastrointestinal Symptom Rating Scale; OFI-8: Oral Frailty Index-8; MNA-HF: Mini Nutritional Assessment-Heart Failure; PHQ-9: Patient Health Questionnaire-9.

**Table S2. The identified differential gut taxa at genus level between gut frailty and non-frailty groups.**

| **Taxonomy** | **Non-gut frialty** | | | **Gut frialty** | | | **Alteration** | **p-Value** | **q-Value** |
| --- | --- | --- | --- | --- | --- | --- | --- | --- | --- |
|  | **Mean** | **Variance** | **Stderr** | **Mean** | **Variance** | **Stderr** |  |  |  |
| g__Methanobrevibacter | 0.004665 | 0.000653 | 0.004665 | 0 | 0 | 0 | Depleted | 0.000999 | 0.00999 |
| g__Slackia | 0.000124 | 0 | 0.000092 | 0 | 0 | 0 | Depleted | 0.000999 | 0.00999 |
| g__Coprobacter | 0.000302 | 0.000003 | 0.000302 | 0 | 0 | 0 | Depleted | 0.000999 | 0.00999 |
| g__unidentified_Prevotellaceae | 0.065057 | 0.012361 | 0.020299 | 0.001659 | 0.000055 | 0.001353 | Depleted | 0.000999 | 0.00999 |
| g__unidentified_Bacteroidales | 0.000114 | 0 | 0.000114 | 0 | 0 | 0 | Depleted | 0.000999 | 0.00999 |
| g__Sarcina | 0.000106 | 0 | 0.000106 | 0 | 0 | 0 | Depleted | 0.000999 | 0.00999 |
| g__Coprococcus | 0.011117 | 0.000127 | 0.00206 | 0.001805 | 0.000019 | 0.000794 | Depleted | 0.000999 | 0.00999 |
| g__Lachnoclostridium | 0.010139 | 0.000077 | 0.001599 | 0.066801 | 0.005603 | 0.013666 | Enriched | 0.000999 | 0.00999 |
| g__Clostridioides | 0 | 0 | 0 | 0.001772 | 0.00008 | 0.001633 | Enriched | 0.000999 | 0.00999 |
| g__Faecalibacterium | 0.067965 | 0.003509 | 0.010815 | 0.022682 | 0.000951 | 0.005631 | Depleted | 0.000999 | 0.00999 |
| g__Dialister | 0.029013 | 0.002735 | 0.009548 | 0.000991 | 0.00001 | 0.000586 | Depleted | 0.000999 | 0.00999 |
| g__Sneathia | 0.001066 | 0.000034 | 0.001066 | 0 | 0 | 0 | Depleted | 0.000999 | 0.00999 |
| g__Moryella | 0.000199 | 0 | 0.000059 | 0.000027 | 0 | 0.000011 | Depleted | 0.001998 | 0.017125714 |
| g__unidentified_Enterobacteriaceae | 0.015243 | 0.000703 | 0.004839 | 0.13081 | 0.034775 | 0.034046 | Enriched | 0.001998 | 0.017125714 |
| g__unidentified_Christensenellaceae | 0.005688 | 0.000107 | 0.001888 | 0.000535 | 0.000003 | 0.000309 | Depleted | 0.002997 | 0.0224775 |
| g__Sutterella | 0.001687 | 0.000037 | 0.001109 | 0.000003 | 0 | 0.000003 | Depleted | 0.002997 | 0.0224775 |
| g__unidentified_Oscillospiraceae | 0.021023 | 0.000657 | 0.00468 | 0.005215 | 0.000228 | 0.00276 | Depleted | 0.003996 | 0.02664 |
| g__Negativibacillus | 0.003606 | 0.000064 | 0.001456 | 0.000236 | 0.000001 | 0.00016 | Depleted | 0.003996 | 0.02664 |
| g__Mitsuokella | 0.001052 | 0.000017 | 0.000758 | 0 | 0 | 0 | Depleted | 0.004995 | 0.031547368 |
| g__Prevotella | 0.02535 | 0.00885 | 0.017176 | 0.000595 | 0.000006 | 0.000438 | Depleted | 0.005994 | 0.034251429 |
| g__unidentified_Ruminococcaceae | 0.003782 | 0.00005 | 0.00129 | 0.00061 | 0.000002 | 0.000229 | Depleted | 0.005994 | 0.034251429 |
| g__Marvinbryantia | 0.000306 | 0 | 0.0001 | 0.000049 | 0 | 0.000029 | Depleted | 0.006993 | 0.038143636 |
| g__Acidaminococcus | 0.001487 | 0.000028 | 0.000968 | 0 | 0 | 0 | Depleted | 0.007992 | 0.041697391 |
| g__Dorea | 0.011647 | 0.000081 | 0.001643 | 0.005391 | 0.000084 | 0.001673 | Depleted | 0.00999 | 0.04995 |
| g__Alistipes | 0.018832 | 0.000831 | 0.005264 | 0.005289 | 0.000125 | 0.002038 | Depleted | 0.010989 | 0.0527472 |
| g__Collinsella | 0.015664 | 0.000343 | 0.003382 | 0.004684 | 0.000191 | 0.002526 | Depleted | 0.011988 | 0.05328 |
| g__Ligilactobacillus | 0.000969 | 0.000019 | 0.000791 | 0.01614 | 0.001571 | 0.007237 | Enriched | 0.011988 | 0.05328 |
| g__Butyricicoccus | 0.004452 | 0.000025 | 0.000913 | 0.001972 | 0.000008 | 0.000509 | Depleted | 0.015984 | 0.068502857 |
| g__Agathobacter | 0.029037 | 0.001963 | 0.008089 | 0.007703 | 0.000581 | 0.004401 | Depleted | 0.01998 | 0.082675862 |
| g__Enterococcus | 0.000374 | 0.000002 | 0.000241 | 0.022258 | 0.008275 | 0.016608 | Enriched | 0.020979 | 0.083916 |
| g__Veillonella | 0.001724 | 0.000017 | 0.00075 | 0.025527 | 0.00429 | 0.011958 | Enriched | 0.022977 | 0.088943226 |
| g__Paludicola | 0.000295 | 0.000002 | 0.000224 | 0.000004 | 0 | 0.000003 | Depleted | 0.024975 | 0.09365625 |
| g__Lactococcus | 0.000001 | 0 | 0.000001 | 0.000101 | 0 | 0.000078 | Enriched | 0.028971 | 0.102250588 |
| g__Megasphaera | 0.007511 | 0.000486 | 0.004025 | 0.000094 | 0 | 0.000091 | Depleted | 0.02997 | 0.102754286 |
| g__Oscillibacter | 0.000189 | 0 | 0.000068 | 0.000046 | 0 | 0.000022 | Depleted | 0.031968 | 0.10656 |
| g__unidentified_Clostridia | 0.014431 | 0.000762 | 0.005039 | 0.004585 | 0.000109 | 0.001903 | Depleted | 0.036963 | 0.11988 |
| g__Desulfovibrio | 0.001166 | 0.000007 | 0.00049 | 0.000137 | 0 | 0.000094 | Depleted | 0.040959 | 0.128871 |
| g__unidentified_Flavobacteriaceae | 0.000128 | 0 | 0.000113 | 0 | 0 | 0 | Depleted | 0.042957 | 0.128871 |
| g__Flavonifractor | 0.001088 | 0.000005 | 0.000391 | 0.003265 | 0.000027 | 0.000953 | Enriched | 0.042957 | 0.128871 |
